# Modeling and prediction of methanol air release from bleached chemi-thermo mechanical pulp board†

**DOI:** 10.1039/c8ra02114g

**Published:** 2018-05-08

**Authors:** Yu Zhang, Xin-Sheng Chai, Liulian Huang, Lihui Chen, Hui-Chao Hu, Ying-Xin Tian

**Affiliations:** College of Material Engineering, Fujian Agriculture and Forestry University Fuzhou 350002 China fafuclh@163.com hhc_huichao@163.com +86-591-83789495 +86-591-83715175; State Key Laboratory of Pulp and Paper Engineering, South China University of Technology Guangzhou 510641 China

## Abstract

This paper reports on the modeling, prediction and evaluation approaches of methanol release from bleached chemi-thermo mechanical pulp (BCTMP) board during storage. A pseudo-first order desorption kinetics model of methanol release was established for describing the desorption behavior of methanol from BCTMP, *i.e.*, 
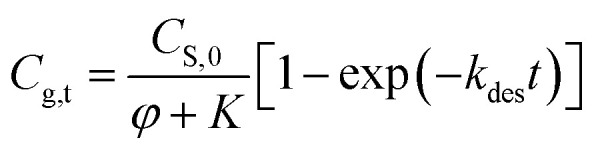
, in which the desorption constant (*K*) and rate constant (*k*_des_) were well described by van't Hoff and Arrhenius equations. Based on the simulation experiments at various temperatures, the desorption activation energy of methanol and its adsorption enthalpy is calculated and is 53.7 and −86.2 kJ mol^−1^ K^−1^, respectively. With the developed model, the risk of methanol release for the storage of BCTMP board can be examined by either the time-dependent kinetics model or a two-step thermodynamic approach using the equilibrium concentration of methanol in indoor air. This paper provides a valuable tool to assess the risk of methanol release for the paper industry and related warehouse departments.

## Abstract 2

This paper reports on the modeling, prediction and evaluation approaches of methanol release from bleached chemi-thermo mechanical pulp (BCTMP) board during storage.

## Graphical abstract

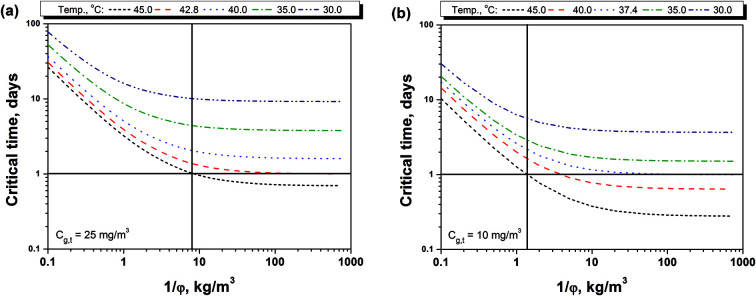

## 1. Introduction

Methanol is a volatile organic compound and toxic to human beings if it is absorbed by ingestion, inhalation, skin contact or eye contact. It is because formaldehyde and formic acid or formate salts, the metabolized products from methanol, are poisonous to the central nervous system and may result in blindness, coma, and even to death.^1,2^ Regulated by United States Occupational Safety and Health Administration and European Union Information Agency for Occupational Safety and Health, the permissible exposure limit-time weighted average (PEL-TWA) in workplace air for methanol is 260 mg m^3^ (ref. 3 and 4) or even lower (10 mg m^−3^ in California).^5^ In China, the PEL-TWA and the permissible concentration-short term exposure limit (PC-STEL) of methanol are 25 and 50 mg m^−3^, respectively.^6^ Therefore, it is very important to control the methanol concentration to a safe level in workplace air in many related industries.

There is a large amount of methanol generated by the pulp and paper industry, mainly from the pulping process. It is because the methoxyl groups on lignin and hemicelluloses in biomass lignocellulosic materials can react with hydroxide to form methanol during alkaline related pulping,^7–9^ oxygen delignification,^10,11^ and bleaching processes^12,13^ through the alkaline hydrolysis and/or free radical promoted demethylation reactions. Extensive studies were conducted in the late 90 s on methanol formation during alkaline pulping processes.^7–9^ The results showed that hardwood pulping could produce more methanol than softwood pulping, in which the methanol contents in the pulping spent liquors at the end of cooking are about 1500 and 1050 mg L^−1^, respectively. The maximum amount of methanol generated in the pulping process could be up to 11.2 kg ton^−1^ pulp.^7^ The pulping mode and pulping catalyst also affect the amount of methanol generated during the pulping processes, in which soda pulping would generated more methanol than kraft pulping and the addition of anthraquinone (as the catalyst) in soda or kraft pulping process could reduce the methanol formation.^7^ In our previous work, we found that the amounts of methanol generated in the oxygen delignification process were in the range of 0.16 to 3.6 kg ton^−1^ pulp, which mainly depends on the lignin contents in the unbleached pulps.^11^ Because methanol can be adsorbed by pulp to some extent, it is very difficult to be completely removed from pulps by the washing process. As a result, there could be a significant amount of methanol remaining in the pulps and its related final products, *e.g.*, paper and paper board.

Recently, we conducted an investigation on the amount of methanol remaining in different pulp and paper products, such as bleached chemi-thermo mechanical pulp (BCTMP), bleached kraft pulp, cup paper, and tissue paper.^14,15^ The results showed that the amount of methanol in BCTMP could be up to ∼3000 mg kg^−1^, about 10 times higher than that in the chemical pulps (277 mg kg^−1^). The amount of methanol remaining in the cup paper and tissue paper were 132 and 341 mg kg^−1^, respectively.

Because of its high content in BCTMP, the methanol emission from the board and the related products (*e.g.*, packaging paper) during the storage becomes a serious safety issue and must be considered. Therefore, it is necessary to have a kinetic model that is able to predict the amount of methanol released from BCTMP board to indoor air, from which the risk assessment and the storage strategy for the products can be conducted. Unfortunately, there is lack of work reported in the literatures on this subject.

The objective of this work was to demonstrate a kinetic and thermodynamic approach for predicting the methanol release behavior from the BCTMP board (the worst case) to indoor air in a closed storage space. The simulating experiment with a temperature induced method will be conducted, aiming to obtain the key kinetic and thermodynamic parameters. Finally, two risk evaluation methods for methanol release from paper products into indoor air were proposed.

## 2. Materials and methods

### 2.1 Chemicals and materials

Methanol was analytical grade and purchased from Sigma Aldrich. A 1050 mg L^−1^ of standard methanol stock solution was prepared by adding 1.050 g of pure methanol to 1.0 L of deionized water. A set of standard methanol solutions with various concentration (150, 210, 350 and 525 mg L^−1^) were prepared by diluting the standard methanol stock solution with deionized water. The maple BCTMP sample, obtained from a pulp mill, was kept in a sealed double-layers polyethylene bag after air-dried in a well-ventilated room at ambient temperature. After 48 hours, the moisture content in BCTMP was measured using TAPPI Test Method (T412om-02 “Moisture in pulp, paper and paperboard”).^16^

A set of handsheets with different basis weights (weight per unit area), from 40 to 200 g m^−2^, was prepared using a semiautomatic handsheet mold (Messmer Instruments Ltd, Model 255, UK), according to TAPPI Method (T205 sp-02 “Forming handsheets for physical tests of pulp”).^17^ All handsheets were cut as one-centimeter-wide and an appropriate length for getting the desired weights.

### 2.2 Methanol release measurement

The measurement of methanol released from the samples was conducted by headspace gas chromatography (HS-GC). The HS-GC system included an automatic headspace sampler (DANI HS 86.50, Italy) and a GC (Agilent GC 7890A, US) that equipped with a flame ionization detector and a DB-5 capillary column (30 m × 0.32 mm × 0.25 μm). The headspace sampler operating conditions were as the follows: strong shaking mode; sample loop temperature = 125 °C; transfer line temperature = 130 °C; pressurization pressure = 2.0 bar; carrier gas pressure = 1.5 bar; vial pressurization time = 15 s; sample loop fill time = 20 s; and transfer time = 20 s. The GC was operated at a temperature of 30 °C with nitrogen carrier gas (flow rate = 3.8 mL min^−1^).

A 0.5 g of BCTMP handsheet was added in a headspace sample vial. After sealing the vial with a PTFE/silicone septum and aluminum cap, it was immediately placed in the headspace sampler for the equilibration at desired temperature (70, 80, and 90 °C) and time (1–300 min). Then, the air phase in the vial was automatically withdrawn by headspace sampler and transferred to GC system for methanol analysis. The GC signals of methanol in air phase were calibrated using the standard curve obtained by a full evaporation headspace testing (at an oven temperature of 100 °C for 5 min) on a set of standard solutions (volume = 5 μL).

### 2.3 Measurement for solid-air partition coefficients of methanol

The partition coefficients of methanol between the BCTMP's handsheet and air phase were detected at temperatures from 70 to 120 °C using a solvent-assisted headspace analysis method reported previously.^18^

## 3. Results and discussion

### 3.1 Modeling of methanol release from BCTMP board

Fundamentally, the release of methanol from paper products to air includes two aspects, *i.e.*, the partition equilibration thermodynamics and desorption kinetics. The partition equilibrium of methanol between paper and air phases at the storage conditions determines the maximum concentration of methanol in indoor air released from paper products, which also has the effect on the driving forcing of methanol desorption.

#### 3.1.1 Partition equilibrium of methanol in paper-air phases

When placing the paper product in a closed room or container, there is a partitioning equilibration of methanol between paper (solid) and air phase. At the equilibrium state, the henry's adsorption constant (*K*) can be used to describe the partitioning equilibrium of methanol, and that is a function of the methanol contents in paper and in air phases,^19^*i.e.*,1a
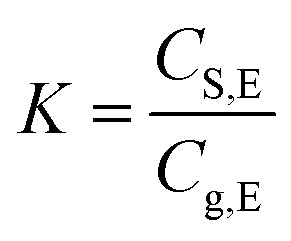
or

1b *C*_S,E_ = *KC*_g,E_

All symbols, definitions, and units have been listed in Table 1.

**Table 1:** Symbols, definitions and units

Symbol	Definition	Unit
*K*	Partitioning equilibrium constant of methanol between paper and air	m^3^ kg^−1^
*C*_S,0_	Initial content of methanol in paper products	mg kg^−1^
*C*_S,t_	Content of methanol in paper products at storage time of ‘*t*’ minute	mg kg^−1^
*C*_S,E_	Content of methanol in paper products at equilibration state	mg kg^−1^
*C*_g,t_	Content of methanol in air phase at storage time of ‘*t*’ minute	mg m^−3^
*C*_g,E_	Content of methanol in air phase at equilibration state	mg m^−3^
*m*_0_	Mass of the paper products added in storage place	kg
*V*_T_	Total volume in the closed storage place	m^3^
*V*_S_	Volume of paper products in the closed storage place	m^3^
*φ*	Ratio of air phase volume to paper mass (storage density)	m^3^ kg^−1^
*k*_des_	Rate constant of methanol release	min^−1^
*k*_des0_	Pre-exponential factor of rate constant of methanol release	min^−1^
*E*_a_des__	Activation energy of methanol release	kJ mol^−1^
*R*	Ideal gas constant	kJ mol^−1^ K^−1^
*T*	Storage temperature	K
*k*_0_	Pre-exponential factor of van't Hoff equation	m^3^ kg^−1^
Δ*H*	Adsorption enthalpy of methanol on paper matrix	kJ mol^−1^

According to mass balance, the methanol total mass in the closed space is equal to the total amount of methanol in the original paper products (*t* = 0 min), *i.e.*, the sum of methanol in the air and paper phase as expressed below:

2 *C*_S,0_*m*_0_ = *C*_S,t_*m*_0_ + *C*_g,t_(*V*_T_ − *V*_S_)

Rearrange eqn (2) to have the relationship between *C*_S,t_ and *C*_g,t_, *i.e.*,3a
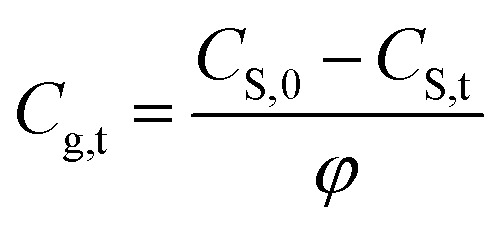
with3b
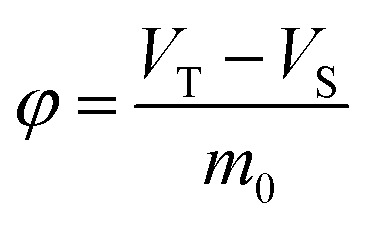


According to eqn (1b) and (3a), the content of methanol released in the air phase at the equilibrium state can be written as,4
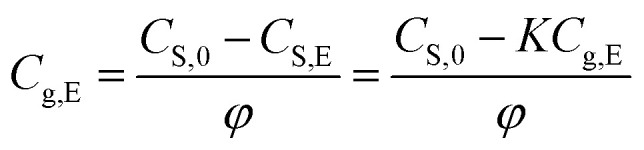


Rearranging eqn (4), we can obtain eqn (5) to calculate the equilibrium concentration of methanol in the air phase, *i.e.*,5
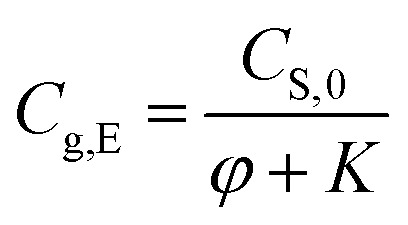


Therefore, the equilibrium concentration of methanol in air phase can be calculated by eqn (5), when the ratio of air phase on solid phase (*φ*) and partition equilibrium constants (*K*) are available.

#### 3.1.2 Desorption kinetics

According to the pseudo-first-order rate equation, the desorption rate of methanol from paper products to indoor air can be written as6a
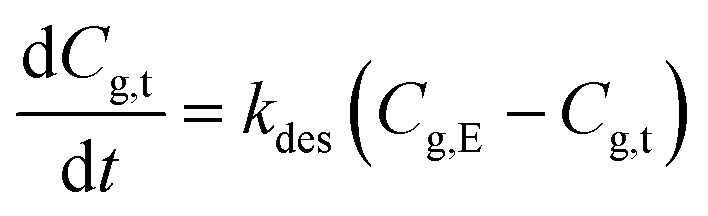
with6b
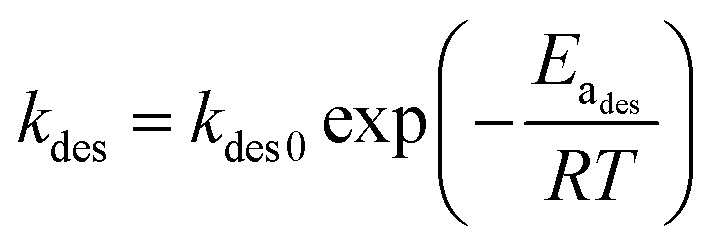


Because the amount of released methanol at time = 0 is negligible, *i.e.*, *C*_g,0_ = 0 mg m^−3^, integrating eqn (6a) to have

7a *C*_g,t_ = *C*_g,E_[1 − exp(−*k*_des_*t*)]

Substituting eqn (5) into eqn (7a) to obtain7b
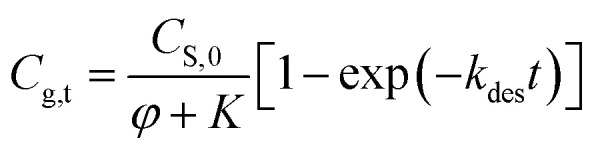


Based on this equation, the amount of methanol release from paper matrix at storage conditions can be predicted when the parameters (*i.e.*, *C*_S,0_, *φ*, *K*, and *k*_des_) are available.

### 3.2 Determination of models parameters for methanol release

In the preliminary experiments, we found that the basis weight of handsheet (40–120 g m^−2^) has a minor effect on the methanol emission rate to indoor air. Moreover, the methanol content in handsheet after three times forming process is nearly at the same value with that in the original BCTMP board. Thus, we used the handsheet with a 120 g m^−2^ of basis weight in the following experiments to obtain the kinetic parameters in eqn (7b) and thermodynamic parameters for calculating the ‘*K*’ value.

In the mill practice, the storage temperature for paper products are basically between 0 to 40 °C, highly depends on the seasonal variation and the location selected. Obviously, the methanol release from paper products at such a temperature range is extremely slow, which could take a few months to achieve the partition equilibration. To accelerate the methanol releasing, we conducted the simulating experiments at a higher temperature range (*i.e.*, 70–90 °C) and established the temperature-involved model, aiming at predicting the methanol concentration in indoor air released from paper products at lower temperature.

#### 3.2.1 Gas–solid partition equilibration constants

**Fig. 1:**
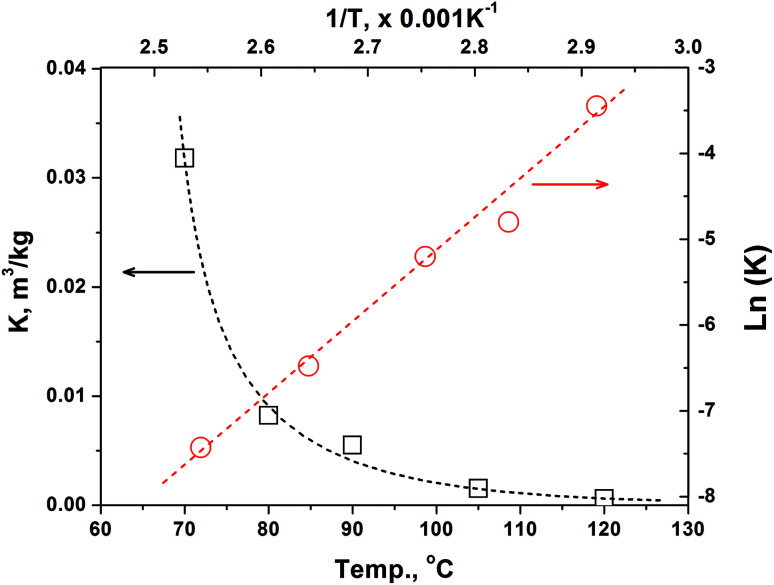
Effect of temperature on the partition equilibrium constants of methanol between BCTMP's handsheet and air phase.

Fig. 1 shows the variation of partition equilibrium constants of methanol as the temperature changes. It can be seen that the value of *K*(*C*_S,E_/*C*_g,E_) is exponentially decreased as temperature, indicating that the higher temperature will lead to a higher methanol concentration at the equilibrium. Accordingly, the methanol desorption from paper products to indoor air was accelerated. Based on the van't Hoff equation, eqn (8a), we can get eqn (8b) by applying the logarithm to the equation. Thus a liner relationship between ln(*K*) and the reciprocal of temperature can be established, as shown in Fig. 1. Therefore, the pre-exponential factor and adsorption enthalpy in eqn (8a) or (8b) can be calculated by a best linear fitting, which are listed in Table 2. The excellent correlation coefficient (*R*^2^ = 0.992) and the small variations of *E*_a_ and ln(*k*_0_) indicates that the van't Hoff equation can provide a good prediction for the variation of *K* with the temperature changing.8a
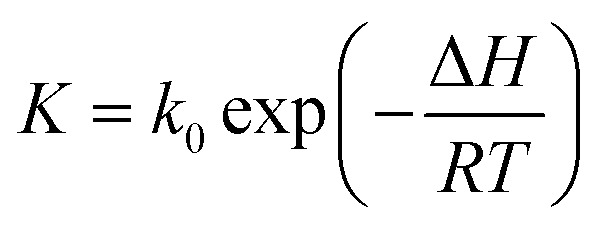
8b
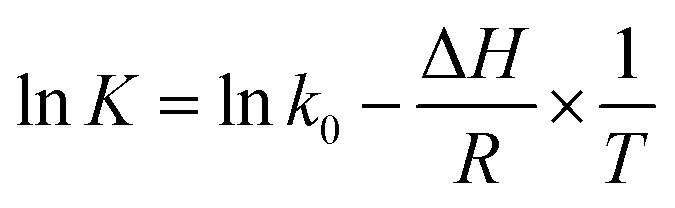


**Table 2:** Adsorption enthalpy and pre-exponential factor obtained from the best-fitting of eqn (8) on the data shown in Fig. 1

	Δ*H*, kJ mol^−1^	ln(*k*_0_ min)
Value	−86.2	−33.9
Error^a^	±6.2	±2.1

a At the confidence level of 95%.

#### 3.2.2 Rate constant and kinetic parameters for methanol release

**Fig. 2:**
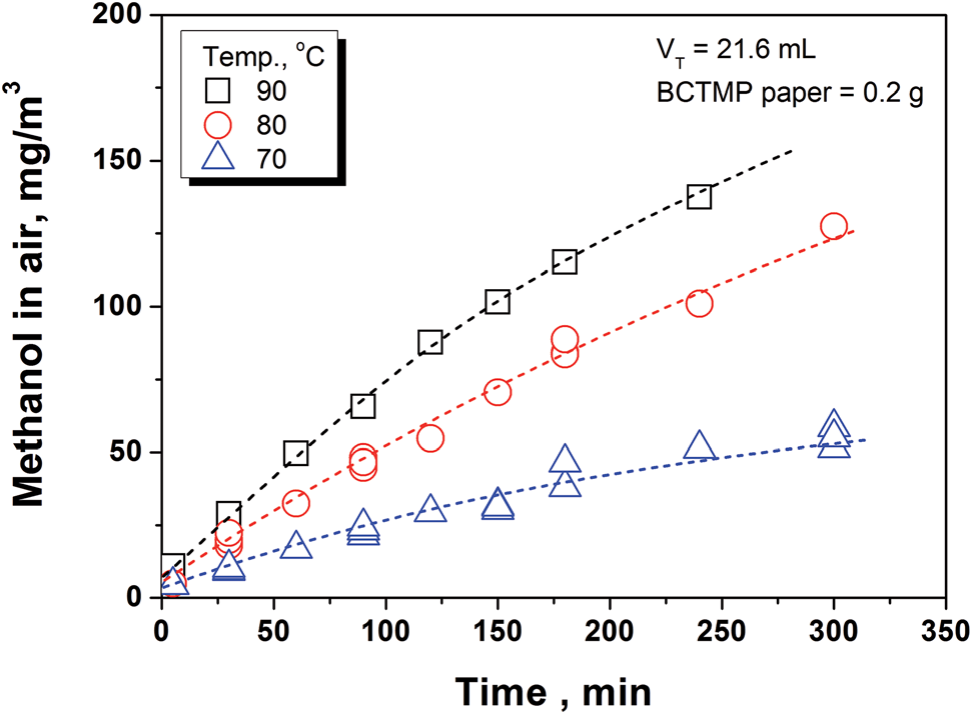
Variation of methanol concentration in gaseous phase at different times and temperatures.

Fig. 2 shows the variation of methanol concentration in the air phase during 5 h's storage at the given temperature range. It can be seen that the higher temperature accelerates the methanol release from the paper matrix. Combining the experimental data shown in Fig. 2, eqn (7b), with the value of the parameters listed in Table 2, the rate constants of methanol release at different temperature (*k*_des_) can be obtained. Using these rate constant data and excel program solving method, the activation energy and pre-exponential factor of methanol release is calculated by eqn (6b), which has been listed in Table 3. Based on the parameters listed in the above tables and eqn (5), (7b) and (8a), the equilibrium concentration of methanol in indoor air and its real-time release amount from paper products to indoor air at various temperatures and storage density of paper product can be predicted.

**Table 3:** Rate constant, activation energy and pre-exponential factor of methanol release

Temp., °C	*k*_des_, min^−1^	*R* ^2^	*E*_a_des__,^a^ kJ mol^−1^	ln(*k*_des0_ min)
70	9.23 × 10^−6^	0.963	53.7 ± 8.57	7.26 ± 2.92
80	1.81 × 10^−5^	0.978		
90	2.59 × 10^−5^	0.977		

a With a standard deviation at the confidence level of 95%.

### 3.3 Rapid risk evaluation of methanol release with gas–solid equilibration

**Fig. 3:**
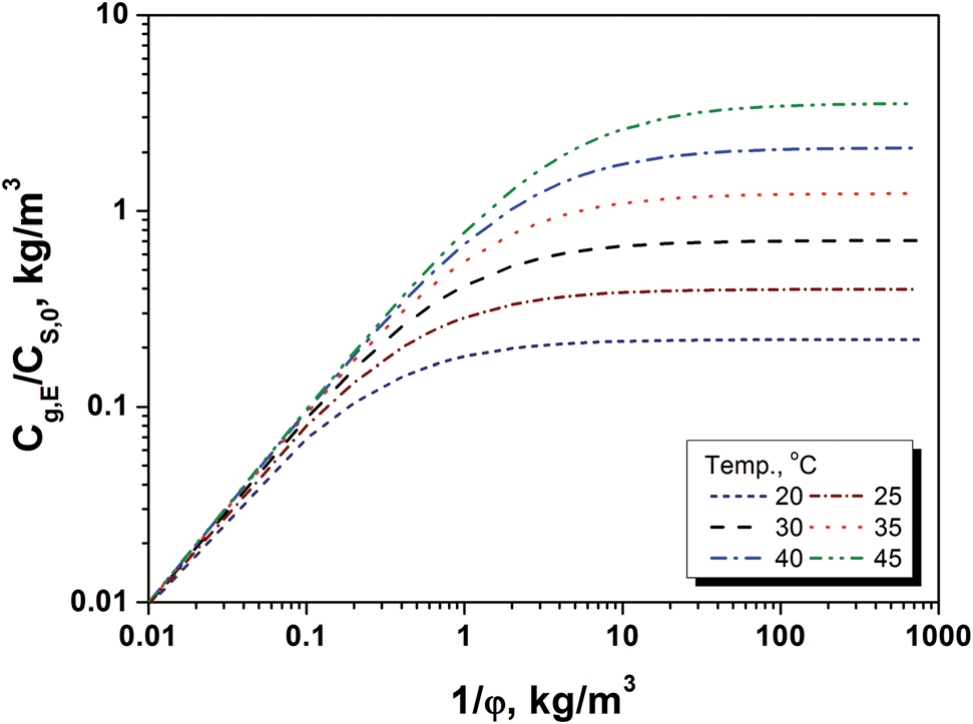
Dependence of *C*_g,E_/*C*_S,0_ on storage density (1/*φ*) at different temperatures.

Since the equilibrium concentration of methanol (*C*_g,E_) is the maximum concentration of methanol in gaseous phase with a sufficient long release time, it can be used as an index for quickly judging if the amount of methanol release will reach at the risk level to the workers at a given storage conditions. Basically, the fast judgment can be finished by a two-step approach that includes calculation of *C*_g,E_/*C*_S,0_ and determination of the methanol content in paper products. Using this BCTMP board as example, firstly, the variation of *C*_g,E_/*C*_S,0_ as 1/*φ* at several temperatures ranged from 20 to 45 °C could be obtained using eqn (5) and (8a). Fig. 3 shows the relationship between *C*_g,E_/*C*_S,0_ and storage density at various temperatures for this BCTMP board used in this paper, which indicate that the equilibrium (maximum) concentration of methanol increases linearly at first, and then levels off as storage density of BCTMP board in air (1/*φ*). The storage density of transition zone and the maximum value of *C*_g,E_/*C*_S,0_ increase as the indoor temperature increasing. It means that the higher indoor temperatures will result in the higher equilibrium concentration of methanol in indoor air.

For example, if the indoor temperature is below 30 °C, then the maximum equilibrium concentration of methanol will be lower than 0.7 times of the methanol content in the selected BCTMP board. For those BCTMP board with lower methanol content (*e.g.*, <371 mg kg^−1^), the methanol concentration in the indoor air will be always within the safe range regulated by EU-OSHA and United State OSHA (260 mg m^−3^).^3,4^ Thereby, the thermodynamic equilibrium can directly evaluate the risk of methanol releasing.

### 3.4 Prediction of methanol content in air released from BCTMP board

The risk of methanol release in the working place can be evaluated by calculating the critical time to reach the PEL-TWA of methanol. Here, we used 25 mg m^−3^ (China) and 10 mg m^−3^, regulated in China and California,^5,6^ as the reference criteria in the critical time calculations, in which the designated storage temperatures were ranged from 30 to 45 °C. By rearranging eqn (7b), the equation for calculating the critical time can be expressed as below, in which the values of *k*_des_ and *K* can be calculated using eqn (6b) and (8a).9
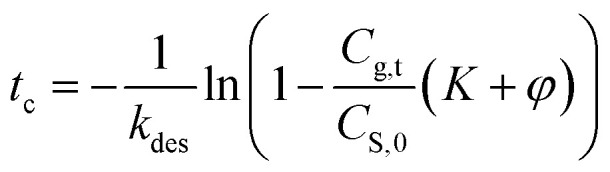


**Fig. 4:**
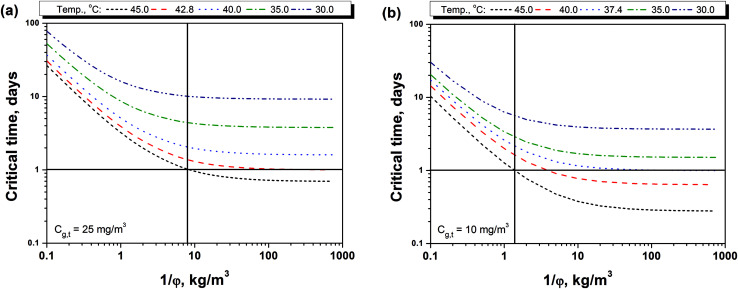
Critical time for reaching the limitations on indoor methanol contents of (a) 25 mg m^−3^ and (b) 10 mg m^−3^ with various temperatures and the storage densities.

Fig. 4(a) and 3(b) shows the relationship between the storage density (1/*φ*) and the critical time (*t*_c_) to reach the PEL-TWA of methanol. It can be seen that the critical time decreased firstly fast and then slowly as storage density at various storage temperatures. The storage density at turning point also increases as storage temperature increasing. Moreover, the effect of storage temperature is significant on the critical time, and the higher temperature will result in a shorter critical time. Fig. 4(a) also shows that the critical time for a 25 mg m^−3^ of PEL-TWA is always longer than 24 hours no matter how the storage density is as far as the temperature is below 42.8 °C. For the PEL-TWA of methanol (10 mg m^−3^), as shown in Fig. 4(b), the methanol in indoor air will be safety for the BCTMP board at all storage density as far as the temperature is below 37.4 °C. In addition, when the storage density is lower than 8 kg m^−3^ or 1.5 kg m^−3^, the methanol content in indoor air will be below the level of PEL-TWA regulated in either China or California at any common storage temperature lower than 45 °C.

## 4. Conclusion

We have developed a pseudo-first order desorption kinetic model for predicting the methanol release to indoor air for the BCTMP board during the storage conditions. The calculated results by this model showed that the methanol content in air within 24 hours in a closed space for the storage was basically below the permissible exposure limits. For a rigorous PEL-TWA in California (10 mg m^−3^) and China (25 mg m^−3^), the storage temperature of BCTMP's board in a warehouse should be lower than 42.8 and 37.4 °C, respectively. The information shown in the present paper could be a safety guidance to the manufacturer and wholesaler for safely storing BCTMP's board. Furthermore, the proposed approaches provided an important referential value for evaluating the methanol release from other paper board or related products.

## Conflicts of interest

The authors declare no competing financial interest.

## Supplementary Material

RA-008-C8RA02114G-s001
