# Supplementary material for: Modeling and prediction of methanol air release from bleached chemi-thermo mechanical pulp board
Source: RSC Adv. 2018 May 8;8(30):16690–5. doi: 10.1039/c8ra02114g (PMC9080308; doi:10.1039/c8ra02114g)
Supplement: RA-008-C8RA02114G-s001 [file RA-008-C8RA02114G-s001.pdf]

Electronic Supplementary Material (ESI) for RSC Advances.
This journal is © The Royal Society of Chemistry 2018

Supplemental Materials

The basis weight (weight per unit area) of BCTMP's handsheet is a potential factor on methanol release. Fig. S1 has shown the effect of basis weight of BCTMP's handsheets on methanol content in air. As illustrated, the GC signal of methanol in gaseous phase of headspace vial does not decreased, but increases linearly as basis weight of handsheet at fixed releasing time. During the process of handsheet forming, the amount of diluting water was a constant. The increase of basis weight results in higher consistency, thereby the amount of methanol desorbed from fibres to diluting water will decrease. Hence, the handsheet with high basis weight produces higher methanol content in handsheet and higher releasing amount of methanol from handsheet to air. Considering the value of slope is only a hundredth of intercept at three selected releasing times, the effect of basis weight of handsheet on methanol release can be ignored. In this paper, the BCTMP's handsheet with a 120 g/m^2 of basis weight was used to the kinetic experiments of methanol release.

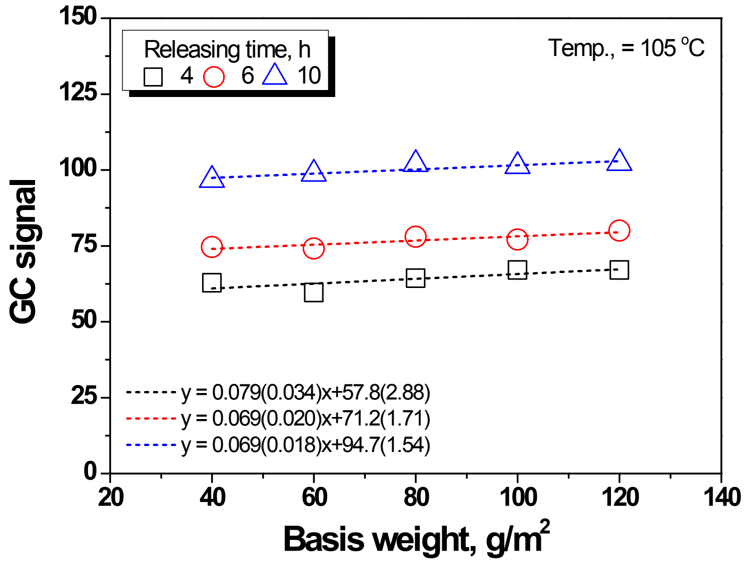

Figure S1 Effect of basis weight of BCTMP's handsheets on methanol releasing
